# Robustness Analysis and Behavior Discrimination in Enzymatic Reaction Networks

**DOI:** 10.1371/journal.pone.0024246

**Published:** 2011-09-27

**Authors:** Alexandre Donzé, Eric Fanchon, Lucie Martine Gattepaille, Oded Maler, Philippe Tracqui

**Affiliations:** 1 UJF-Grenoble 1, CNRS, Laboratoire VERIMAG UMR 5104, 2, Gières, France; 2 UJF-Grenoble 1, CNRS, Laboratoire TIMC-IMAG UMR 5525, DyCTiM and BCM teams, Faculté de Médecine de Grenoble et In3S, Grenoble, France; Hospital for Sick Children, Canada

## Abstract

Characterizing the behavior and robustness of enzymatic networks with numerous variables and unknown parameter values is a major challenge in biology, especially when some enzymes have counter-intuitive properties or switch-like behavior between activation and inhibition. In this paper, we propose new methodological and tool-supported contributions, based on the intuitive formalism of *temporal logic*, to express in a rigorous manner arbitrarily complex dynamical properties. Our multi-step analysis allows efficient sampling of the parameter space in order to define feasible regions in which the model exhibits imposed or experimentally observed behaviors. In a first step, an algorithmic methodology involving sensitivity analysis is conducted to determine bifurcation thresholds for a limited number of model parameters or initial conditions. In a second step, this boundary detection is supplemented by a global robustness analysis, based on quasi-Monte Carlo approach that takes into account all model parameters. We apply this method to a well-documented enzymatic reaction network describing collagen proteolysis by matrix metalloproteinase MMP2 and membrane type 1 metalloproteinase (MT1-MMP) in the presence of tissue inhibitor of metalloproteinase TIMP2. For this model, our method provides an extended analysis and quantification of network robustness toward paradoxical TIMP2 switching activity between activation or inhibition of MMP2 production. Further implication of our approach is illustrated by demonstrating and analyzing the possible existence of oscillatory behaviors when considering an extended open configuration of the enzymatic network. Notably, we construct bifurcation diagrams that specify key parameters values controlling the co-existence of stable steady and non-steady oscillatory proteolytic dynamics.

## Introduction

Nonlinear temporal dynamics, ranging from simple bistable behaviors to oscillatory or even chaotic regimes, play a fundamental role in systems biology. As far as such behaviors are associated with biological functions, a key issue of the biological system analysis is then the ability of going from *qualitative* to *quantitative* dynamical features. Accordingly, the determination of the feasible regions in the parameter space leading to such complex dynamics becomes an important challenge, notably for designing appropriate model-driven experimental strategies. Clearly, the mathematical theory of nonlinear dynamical systems provides methods to identify bifurcations topologies that organize typical asymptotic solutions such as multiple steady-states, limit cycles etc. However, bifurcation theory mostly deals with low dimensional systems controlled by a small number of bifurcation parameters. When dealing with large systems encompassing dozens of parameters, identifying the one or two key bifurcation parameters (or combination of parameters) that crucially affect the system dynamics is not an easy task in general. In addition, the bifurcation theory says nothing about the size and boundaries of the attraction basins associated with each, possibly co-existing, stable asymptotic states. Therefore, such approach does not really meet the requirement of the experimentalist, who needs to know -(i) which parameters or combinations of parameters would affect the regulatory interactions and feedback loops of the considered biological system, and -(ii) to which extent the behaviors that have been identified are robust to exogenous or endogenous perturbations. Indeed, such expectation is central both for understanding how far the system may express a physiological behavior before bifurcating toward a pathological state and for assessing reliable predictions of the system behaviors in different contexts.

Another crucial aspect in biological systems modelling is that the considered parameter values are generally uncertain. Indeed, the preliminary phase of model construction consists in selecting the components, species and reactions to be included in the model, and in the determination of the parameter values (initial conditions, kinetic constants, etc). These values can be obtained from the literature and/or from newly available experimental data. Although the general feeling today is that huge amounts of data are available in data bases, they are not of the type needed to build kinetic models. Most of the time some of the needed parameters have not yet been measured, and those that have been must be used with caution. It is for example recognized that values of kinetic constants from *in vitro* measurements on purified enzymes can be significantly different from the *in vivo* values, due to interactions with other cell components or to sequestration. Also, the values taken from the literature are often heterogeneous (different cell types, different conditions). Measurements obtained on identical cell types placed in supposedly identical conditions can also be qualitatively different for multiple reasons (undetected heterogeneity of cell populations, different batches of antibodies, to name a few). The fact that the amount of available data and information is generally too low with respect to the size of dynamical cell models means that there is not enough constraining information to identify a fully instantiated model. Thus, to represent explicitly the state of knowledge it is best to consider not a single parameter valuation but a *set* of those as discussed in [1]. All the parameter valuations in this set share the property that the associated instantiated models are compatible with available data. This approach is to be preferred to the more common one where uncertain parameters are set to a single numerical value. The arbitrary choice of a representative valuation could potentially lead to unreliable predictions.

Finally, even if we assume that the model is quantitative and properly calibrated, and if the existence of a stable steady-state is known, analyzing transient regimes still remains a challenging issue since bifurcation theory provides no information about the different transient regimes (trajectories) leading to this steady-state. In this context, quantification of robustness is especially required. Thus, practically, the values taken by different protein concentrations at intermediate times may be more informative than their asymptotic state. To perform a quantitative analysis of transient behaviors, one has to resort to simulation and different post-processing algorithms which are generally tailored specifically for each model and situation and thus cannot be reused from one modelling problem to another.

In this paper, we propose a combined approach that takes place after the model construction. Then, we are left with two types of questions for which we would want quantitative, i.e. more than yes or no answers. The first one is : what are the effects of parameter variations on the model behavior(s) ? This problem is addressed by *robustness analysis* and has been dealt with in numerous works (e.g. [2]–[7]). It is also strongly related to *sensitivity analysis* [8], [9]. Since the uncertainties around the parameters cannot always be considered as small, one has to consider (or combine as in [10]) both *local* (i.e. related to one trajectory) and *global* (set of trajectories) approaches to this problem.

The second question, which we dub *behavior discrimination*, is less frequently treated explicitly in the systems biology literature. It can formulated as : when “exploring” the uncertain parameter space, what type of behaviors does the model exhibit and how to identify them ? Following recent trends to apply formal methods to systems biology [11]–[13], we advocate here the use of temporal logics [14] to specify the properties of interest. The advantage of using formal specification languages is that they are simpler to use than plain programming languages and more rigorous, i.e, less error-prone. Moreover, they come with automatic methods to check that a model satisfies a property [15]. In the best cases, the task of the user can be simply reduced to specifying in a given formal language the behavioral properties of interest. Initial approaches to apply formal methods to systems biology attempted to leverage the most advanced techniques in the domain that were developed for discrete transition systems. Thus, they require that the model is either already of this type (e.g. a boolean networks [16]) or that it is abstracted into such a model. In this work, we avoid this additional modelling and abstraction step (which can be intricate to execute properly and may introduce serious scalability issues through the well known state-explosion problem [15]) by proposing specifications and tools that apply directly to simulations of general models of ordinary differential equations (ODEs). We propose a language which can be used to discriminate qualitative properties such as those studied by nonlinear dynamical systems theory (stability, limit cycles, etc) as well as quantitative properties of transient behaviors.

More specifically our methodological contribution is as follows:

1. We propose a language, based on the intuitive syntax of the *signal temporal logic* (STL, [17]), to express in a rigorous manner arbitrarily complex properties. The semantics is both logical (yes/no) and quantitative, meaning that the algorithm for deciding whether or not a trajectory satisfies a property also provides a real number which quantifies the level of satisfaction or violation [18]. This feature automatically captures a notion of local robustness degree;
2. We propose a methodology extending that of [19] for exploring the parameter-space of the system to find the boundaries between the set of parameter values that lead to behaviors that satisfy a given property and those that induce trajectories where the property does not hold;
3. For large numbers of uncertain parameters and/or large domains of uncertain values, we make use of Quasi Monte Carlo methods [20] to estimate the global robustness of the system. For that purpose we exploit on the one hand the Boolean satisfaction of the formula to estimate the relative size of the domain associated with behaviors that satisfy the property, and on the other hand the quantitative satisfaction to provide a global degree of robustness.

We apply our methods to the analysis of enzymatic processes driven by Metalloproteinases (MMPs), a family of enzymes crucially involved in cancer metastasis and angiogenesis [21]. Indeed, MMPs are zinc-dependent endopeptidases that play critical roles in the degradation and, more globally, in the remodeling of extracellular matrices (ECM). This rather large family of enzymes includes not only diffusible MMPs, but also membrane-type matrix metalloproteinases (MT-MMPs) that have emerged as key enzymes in cell biology [22].

**Figure 1:**
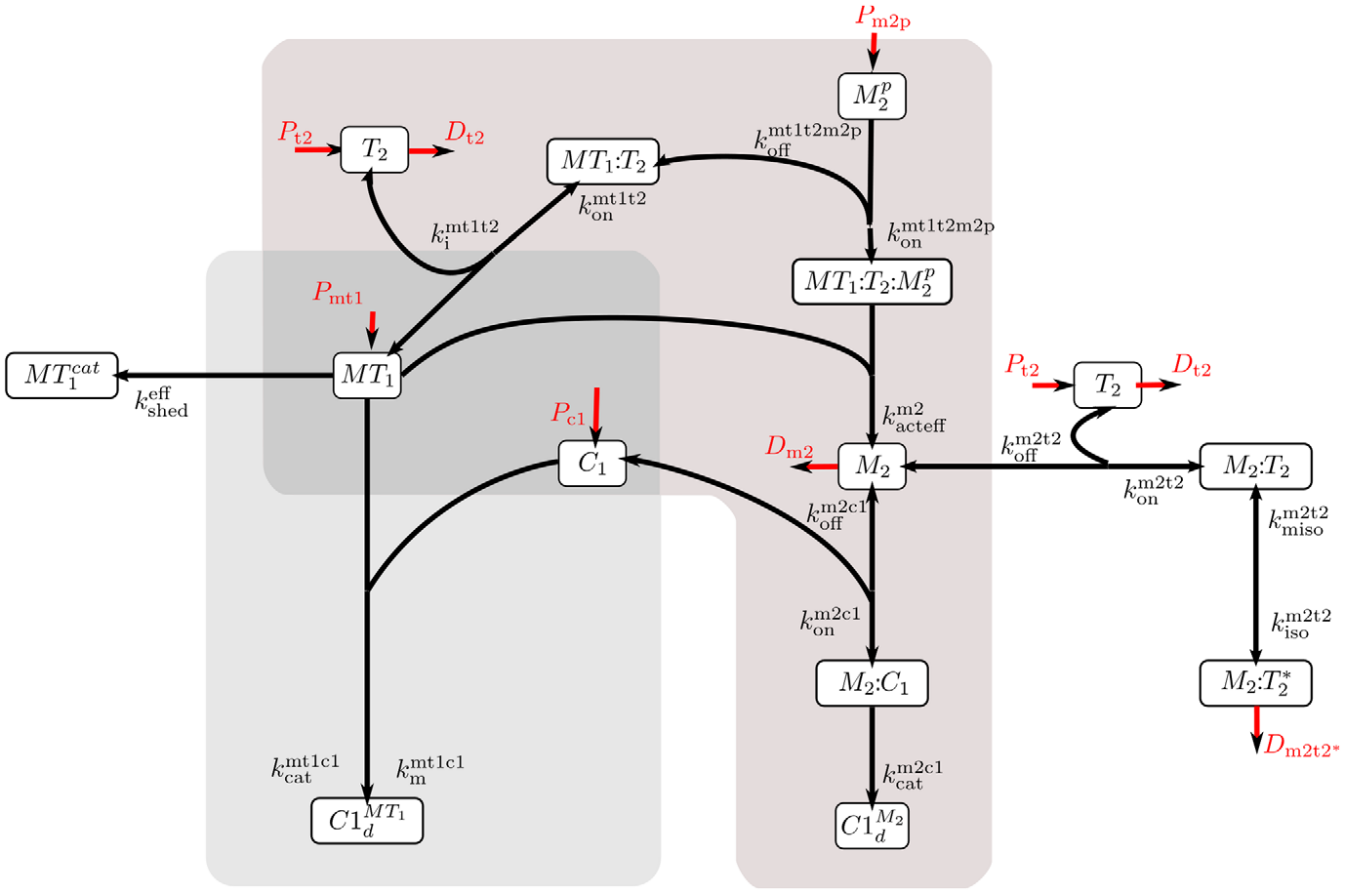
Enzymatic network. In the first part of the Results section, we set the production and degradation parameters to 0 to study the closed system. The different species are described in Table 1. The two concurrent pathways for the degradation of Type I Collagen (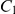) by MT1-MMP (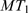) and MMP2 (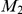) are highlighted. Notice the ambiguous role of TIMP2 (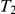) in both of these pathways.

The importance of one of those, MT1-MMP, has been particularly highlighted. Indeed, its interaction with the tissue inhibitor of metalloproteinase-2 (TIMP2) is required for the production of another matrix metalloproteinase, the MMP2, which is synergistically involved with MT1-MMP in ECM degradation. More precisely, the latent or zymogen form of this enzyme, pro-MMP2, is activated on the cell surface through the formation of a ternary complex of active MT1-MMP and TIMP2 bound to pro-MMP2. One hallmark of this biochemical network complexity is the still unsolved TIMP2 paradox: this inhibitor of both MT1-MMP and MMP2 activity is also required for pro-MMP2 activation leading to MMP2 formation. Taken all together, this double enzymatic cascade with feedback loops defines a complex biochemical network (see Figure 1) in which membrane-bound and diffusible MMPs are regulated by a common inhibitor.

This biochemical network is used as a case study for evaluating the new methodological approaches developed in this paper. Taking benefit of the recent and well documented model of type I collagen proteolysis by MMP2 and MT1-MMP in the presence of TIMP2 proposed by Karagiannis and Popel [23], [24], we propose a systemic analysis of the biochemical network properties in two successive stages. The first one is restricted to the analysis of the closed enzymatic network configuration, as in the original work of [23]. The second part extends this analysis to an open enzymatic network configuration, with production fluxes of some enzymes coming into play. Finally, we discuss the contribution of our approach as a powerful technique for a refined and systematic analysis of the dynamical properties of complex enzymatic networks.

## Methods

### Methodology overview

Our formal framework, implemented in the tool Breach [25], consists of the following objects:

- A *parameterized dynamical system*, hereafter simply named *the system*, which is given by a set of differential equations and associated parameters;
- *Uncertain parameter sets (or simply parameter sets)*, each of which consisting in a nominal valuation of the system parameters and a range of possible values centered around this nominal valuation to account for imprecision or uncertainty;
- *Trajectories* are possible time courses of a fully instantiated dynamical system (i.e., in which all the parameter values and initial conditions are specified); An uncertain parameter set is compatible with multiple trajectories depending on the instantiation of its parameters; the nominal trajectory is the trajectory obtained with the nominal parameters;
- *Quantitative temporal properties* characterizing temporal patterns and interconnexions between behaviors of the system as observed by trajectories. A trajectory can evidently satisfy multiple properties. A parameter set is said to satisfy a property if *all* of its associated trajectories satisfy it;

In this work, we assume that the system is given. A typical analysis using our framework is then as follows. We begin with an a priori uncertain parameter set, i.e., a nominal valuation of the system parameters and initial conditions, together with ranges of uncertainty around this nominal valuation. Then, we formalize an hypothesis or an observation about the system and its variables: a concentration is going to fall under a certain threshold before another one, a reaction flux will reach a steady state after some time, etc. Once such observation or hypothesis has been expressed as a property expressed using our language, our software Breach [25] (see also Text S1), can generate trajectories of the initial parameter set and verify at the same time whether they satisfy the property or not. Thus, two main situations are possible: If the parameter set appears to satisfy the property, then we try to characterize the robustness of this satisfaction. Otherwise, we *split* the initial parameter set into a number of more precise subsets by choosing other nominal values for the parameters. Then, we reconduct the analysis over these refined parameter sets, using either the same property or new ones. This approach is described below in more details.

### Parameterized dynamical system, trajectories and uncertain parameter sets: formal definitions

In this work, the system under consideration represents a biological network of 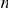 species (enzymes, proteins, complexes etc), which interact in a well stirred environment so that the evolution of their concentrations, noted 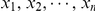, can be described by a set of ordinary differential equations (ODEs) of the form:
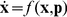
(1)where 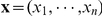 is the *state vector*, 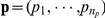 the *parameter vector* and the function 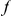 give the rates of the concentrations variations.

We assume that 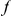 is continuously differentiable on 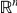 and as a consequence, the system is deterministic: a value for the initial concentrations and the parameters 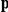 gives rise to only one evolution of the concentrations. Formally, given 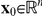 and 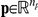, a unique solution 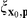 exists for (1) for all time 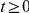. This solution is called a *trajectory* or a *behavior* of the system. For brevity, we will omit 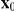 in the notation of trajectories: we write 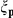 and sometimes only 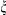 when 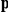 is also fixed in the context (In Breach, initial conditions are actually treated as parameters).

As stated in the introduction, initial conditions and parameters are often not known precisely for biological systems. For this reason, we define the notion of *uncertain parameter sets*. An uncertain parameter set 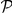 is a set of possible values for 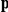 and we are interested in the properties of the trajectories 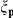 when 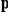 is in 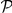. In our implementation, we restricted parameter sets to be hyperboxes (products of intervals) centered on a nominal value. The reason is that such sets are easily sampled or subdivided into subsets of the same nature. Formally 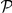 is a pair 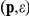 where 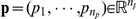 is the nominal valuation of 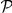 and 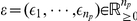 is the range of uncertainty of 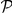. The pair 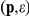 defines 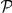 as the hyperbox:
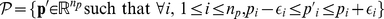
(2)


Every 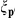 with 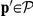 is a trajectory compatible with the uncertain parameter set 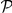.

### Quantitative temporal properties

A trajectory 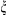 defines a possible time evolution of the 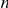 concentration values 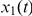, 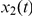, 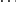, 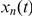 for all time 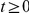. From the quantitative information given by the real numbers 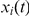, qualitative information can be inferred. E.g., by plotting or processing the data of 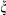 one can observe whether the values converge toward a steady state, monotically increase, oscillate, etc. Of interest can be also transient quantitative information such as the relative values of 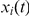 for 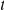 in some time interval and the values of 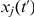 for 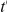 in some later time interval. To extract this type of information, a cost function is usually defined and evaluated through dedicated routines used on 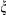 [1], but their implementation can be tedious and error prone. Our framework allows to automate this process by mean of an appropriate language that can express timed and quantitative properties of 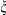 in a compact, intuitive and flexible way. Using this language, we can go from an hypothesis on the system behavior (based on a priori qualitative knowledge from biologists or on observations of experimental data) to a formula 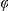 which can be checked rigorously and efficiently on the trajectory 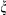. Since the manner we define and use temporal properties constitutes an important part of our contribution, we devote the last part of the methods section to this aspect.

### Local robustness and sensitivity analysis

Once an observation or an hypothesis has been properly expressed by a formula 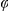, it is important to assert how robustly it holds for a given trajectory 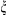 and if the change in parameter values is likely to affect its truth value. Such analysis will be called *local* robustness and sensitivity analysis since we consider only one trajectory, by contrast with *global* robustness and sensitivity analysis where we consider all trajectories compatible with a parameter set 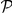, to be discussed in the next section.

We define precisely the notion of local robustness of a property 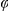 by introducing a *robust satisfaction function* 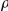. This function takes a formula and a trajectory as inputs and returns a real number:
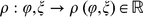
(3)The function has the following fundamental property : 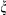 satisfies the property 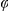 if and only if 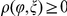. Intuitively, if 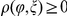, then 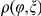 measures how far 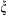 is from violating the property 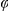.

*Local sensitivity analysis* refers to the study of the influence of a small perturbation of 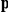 on the trajectory 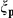. It is measured by the derivative of 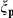 with respect to 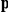: 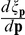, which is called the sensitivity function. Efficient methods exist to compute this derivative alongside with the computation of the trajectory by solving the system (1) extended with additional equations describing the time evolution of the sensitivity function (see [19] for more details). For 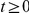, we get 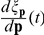 which is an 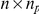 matrix whose 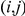 component is 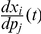. To get an estimate of the relative variation of some variable 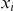 at time 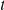 with respect to the relative variation of some parameter 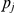, we compute the quantity:
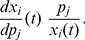
(4)Given a formula 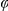, we showed in [18] that if we know the sensitivity function 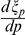, we can, under certain conditions, compute the derivative of 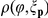 with respect to 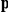 (see Text S2 for additional details). This allows us to define a notion of local sensitivity of a formula 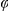 with respect to a parameter 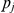 as the relative variation of 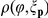 with respect to the relative variation of 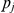:
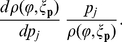
(5)


### Global robustness and sensitivity analysis

#### Reachability analysis

As mentioned above, global robustness refers to the robust satisfaction of a property not only by a trajectory but by all trajectories of an uncertain parameter set 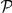. Ideally, one would like to be able to check a property for every trajectories 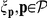. For safety properties, e.g. of the form “the system always avoids a forbidden region of the state space”, this is possible through the computation of an over-approximation of the reachable set 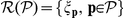 and its intersection with the forbidden region. If this intersection is empty, then 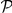 satisfies the formula. The computation of reachable sets has been the topic of steady research efforts during the last two decades (see [26] for recent progress and an illustrative application on biological models). The main difficulty is the dimensionality of the system. Indeed, most of the existing techniques suffer from the curse of dimensionality and quickly become inapplicable as the number of state variables 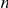 increases. In [19], we present a method, implemented in the tool Breach, which does not suffer from this problem. It approximates the reachable set using a finite number of trajectories and sensitivity functions by hierarchically sampling the uncertain parameter set 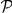. The approach is applicable even for large 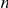 however its precision is sensitive to the size of the uncertain range 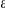. When the number of parameters 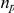 is large and their uncertain range 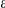 is not negligible, we switch to a Monte Carlo approach.

#### Quasi-Monte Carlo analysis

The idea is to sample 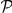 uniformly with 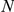 parameter vectors 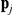, 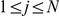 and estimate the frequency of satisfaction of 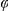 using:
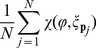
(6)Where 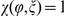 if 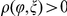 and 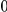 otherwise. If (6) remains constant equal to 1 as 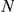 increases, then it is likely that 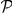 satisfies 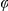. To sample 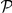 we use *quasi-random* numbers since they are known to have better convergence properties than pseudo-random numbers [20].

The quantity (6) measures a qualitative notion of global robustness by considering for each 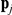 the Boolean satisfaction of 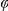, given by 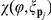. A quantitative estimate can be obtained by averaging directly 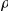 instead:
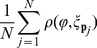
(7)Of course, the interpretation of (7) depends on the definition of the property 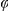. It can be crucial to estimate whether a property that appears to be satisfied for a whole range of values is indeed satisfied with a significant margin of confidence. Finally, *global sensitivity analysis* of the property 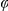 with respect to a parameter 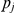 can be quantified by
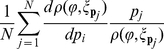
(8)This quantity can be very useful to compare the relative influence of the different parameters on the satisfaction of 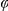 for a whole parameter set 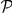.

To compute (6)–8) Breach provides a simple Quasi-Monte Carlo implementation which has been used to produce the results in this paper. It is worth mentioning that it can be improved further with state-of-the-art Quasi-Monte Carlo techniques (see e.g. [27], [28]) in a straightforward way. Other techniques such as the ANOVA decomposition and classical global sensitivity indices reviewed in [8] can also be implemented. Furthermore, the availability of derivatives with respect to parameters for the satisfaction function 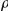 allow to consider the more recent derivative based global sensitivity measures described and advocated in [6], [9].

### Property based refinement of uncertain parameter sets

In this section, we briefly describe how we identify subsets of 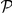 which robustly satisfy a property 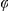. Since 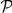 is an hyperbox (see Eq. (2)), it is straightforward to partition it into a regular grid of subsets. For each of these subsets we check whether it robustly satisfies 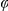. If this is not the case, we iteratively refine it until we find subsets which satisfy or violate the property or which are of insignificant size. The expected result of the overall procedure is a partition of 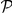 into small subsets around the boundary between satisfaction and violation of 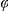 and larger regions where the satisfaction or violation is robust. The method is detailed in [19] for simple properties such as “a given quantity does not go beyond a given threshold” using simulation with nominal parameters and local sensitivity analysis to decide whether to refine a subset or not. Applying the same algorithm using the satisfaction function and its derivative with respect to parameters such as described above, the method is straightforwardly extensible to general temporal properties.

Since one refinement step produces a number of subsets exponential in the number of parameters 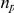, the method can only be applied if we select beforehand a small number of parameters. This selection can be based on a priori knowledge or constraints, or on a sensitivity analysis by choosing parameters which maximize the local sensitivity (4) or global sensitivity (5).

### The temporal logic and its quantitative semantics

We present in this section the syntax and semantics of the logic we use to specify properties.

#### Signal Temporal Logic (STL)

Temporal logic is a special modal logic suited for specifying *properties* of *time-dependent* phenomena. Originally conceived for philosophical purposes by Arthur Prior in the 1960s, it has been exported to the specification and verification of computer systems [14] and ever since played a major role in the verification of *reactive systems* [29], the computer science term for systems that maintain an ongoing interaction with their environment. In this context, the logic allows one to express properties of *sequences of states/events* produced by a concurrent program, for example “two programs will never write simultaneously on the same memory location” or “a program will stay in a given set of states until some event occurs” or “every request of a resource will be eventually followed by granting it” that we view as a generic *response* property.

Once such desired properties have been expressed, they can be automatically verified against individual behaviors generated by the actual system or its simulation. There is an automatic procedure which takes a pair consisting of a property and an execution trace and says whether the behavior satisfies the property. In certain settings it is even possible to prove satisfaction of a property by *all* behaviors generated by a system even if there are infinitely many of them (model-checking). Computer systems are modeled over a discrete state space and discrete logical time. The adaptation of temporal logic to express properties of continuous trajectories requires several modifications, the first being the passage from discrete to dense time as in the logics MTL [30] and MITL [31] which allow one, for example, to refine the response property into “every request is followed by a grant within 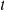 time where 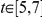 seconds”. The logic that we use is based on the logic STL *signal temporal logic* [17] which augments MITL by predicates (constraints) on the real-valued variables and can express response-like properties in the continuous domain: “if the concentration of 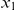 goes above some threshold 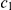 then within 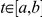 time the concentration of 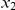 drops below 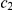
”. Needless to say, the ability to express such properties enriches the vocabulary for describing the behavior of biochemical reactions and complex systems in general.

The monitoring procedure for STL [17] gives a yes/no answer which does not quantify the “strength” of satisfaction/violation. Inspired by several recent works [12], [32]–[36] we defined a *quantitative* semantics for STL [18] which for every trajectory-property pair, gives numbers that indicate how far (in space and time) we are from violation/satisfaction. For the response property it might be the case that within 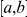 time we reached 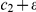 (space robustness) or that we have reached 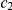 within 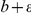 time (time robustness). It is this semantics that we use in the present paper and describe below.

#### STL syntax

A formula 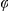 in our logic is constructed from atomic *predicates* which characterize instantaneous properties of a trajectory, combined via *Boolean* and *temporal* operators which relate these properties with respect to one another across different time periods. Formally, a formula is constructed using the following grammar:
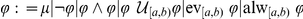
E.g., 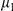, 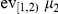, 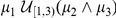, etc, are valid formulas. The terms 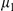, 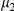, etc, are predicates. A predicate describes a constraint on the state of the system, such as “the concentration of one species 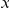 is higher than 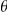
”, where 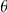 is some given threshold. Formally, 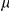 is a generic constraint applied to a trajectory 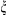 defined as function of a time instant 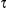. The *canonical form* of 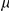 is
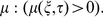
(9)A simple instance corresponding to our example above is
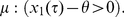
(10)By an abuse of notations we identify the predicate 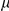 with the function 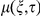 that appears in the left-hand-side of the canonical form of the constraint by which the predicate is defined. Note that, in Breach, the function 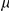 can be quite general and can include nonlinear expressions in 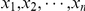, time derivatives (such as 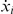) and parameter sensitivities (such as 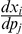).

#### Boolean and temporal operators

To build a formula 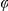, predicates and sub-formulas can be combined using Boolean and temporal operators. Boolean operators include negation 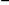, conjunction 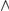 and disjunction 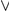 so that, e.g., if 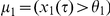 and 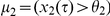 then
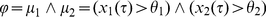
(11)specifies that at time 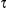, 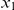 must be above 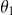 and 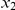 above 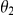.

Using *temporal* operators, one can specify relations between values of the variables at different time instants. The most common temporal operators are *eventually* and *always* (that we abbreviate respectively as “
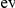
” and “
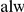
”). The formula 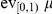 means that the constraint 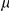 has to be true at least once *before one second* in the future while 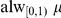 means that 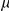 must be satisfied *for all time during one second*. The 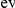 and 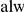 operators are unary operators. There exists also a binary operator named *until*. The formula “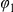 *until* 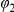
” (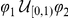) is satisfied if 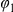 holds continuously until some time before one second when 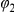 becomes true.

#### Boolean semantics

Formally, we evaluate whether a temporal formula 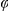 is satisfied or not by a given trajectory 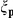 at a time instant 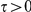. If this is the case, we write
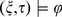
(12)If 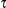 is not specified, it is implicitly 0, i.e., 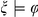 means 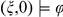. If 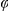 is a predicate 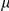, it is satisfied if the constraint associated with 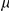 is satisfied by 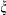 exactly at time 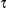, i.e.,
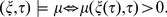
(13)Temporal operators are parameterized by an interval 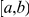 (which is 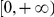 when omitted) and their satisfaction at time 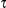 depends on the satisfaction of the subformula during the interval 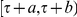:
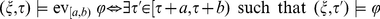
(14)

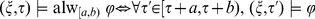
(15)

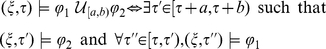
(16)


#### Quantitative semantics

The Boolean semantics given by (13–16) decides whether the trajectory 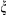 satisfies the formula 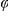 at time 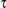 by induction on the structure on the formula. This provides a qualitative interpretation of 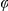. To get a quantitative interpretation, we define a function 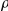 called satisfaction function which takes as arguments 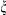, 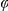 and 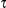 and returns a real number 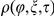 quantifying the degree of satisfaction of 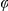 by 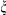 at time 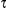. Again, and as we did in the previous Sections, we omit 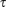 when it is 0: 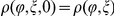.

According to (13), the qualitative semantics associated with a predicate 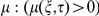 depends on the sign of 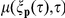. The predicate is true if it is positive and false otherwise. From there, our definition of the quantitative semantics associated with 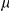 is immediate: it is given by the value 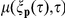:
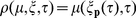
(17)


E.g., for the simple instance (10), it gives 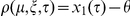.

The quantitative semantics for Boolean and temporal combinations of formulas is defined in such a way that it preserves the property that the sign of 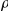 determines the qualitative satisfaction of 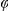. In other words, we define 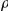 such that it satisfies:
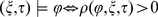
(18)For the negation, it reads:
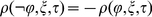
(19)For conjunction and disjunction, we make use of 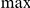 and 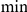 operators:
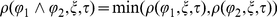
(20)

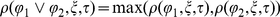
(21)For temporal operators, the quantitative semantics is also achieved using maximum and minimum of the quantitative satisfaction of sub-formulas, with the refinement that we use 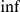 and 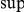 operators to comply with infinite or open finite intervals:
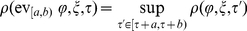
(22)

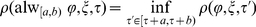
(23)

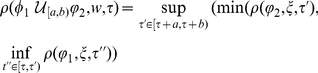
(24)The proof that this definition of 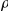 satisfies the constraint (18) can be found in various references, e.g. [35], and an algorithm to compute 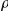 efficiently is described in [18]. We can give an intuitive interpretation of the signification of 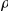 for the “eventually” operator. Recall that for 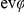 to be satisfied by 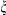 at 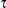, we need a time 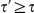 when 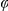 is satisfied. Thus if the supremum of 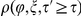 is positive, then there is a time 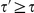 such that 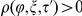, meaning that 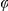 is satisfied for 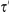 and 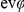 is satisfied for 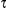. Moreover, 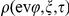 represents the largest satisfaction available for the formula 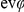 along trajectory 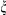. On the other hand, if the supremum of 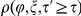 is negative, 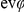 is obviously not satisfied and 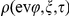 represents how far 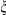 is from satisfying 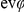. In both cases, 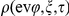 is a fair estimation of the “robustness” of the satisfaction or non-satisfaction of the formula 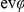 by 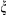 from time 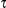. The interpretation for the “always” operator is similar by a simple duality between maximum and minimum operators. Interpreting the “until” operator is trickier because it involves two formulas 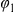 and 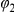. To keep it as simple as possible, 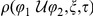 takes its value at a time when the formula 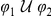 has the highest (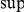) chance to be satisfied (or is satisfied with its highest margin) and at this time picks the weakest (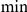) among the satisfactions of the conditions related to 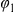 and to 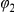. A simple formula illustrates the 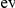 operator at the beginning of the Results Section.

## Results

We applied our methodology to a model of enzymatic network adapted and extended from [23]. A graphical representation of the network is shown on Figure 1 along with the names and description of the different species on Table 1. On Table 2 we provide the corresponding differential equations and on Table 3 the nominal values of the parameters.

**Table 1:** The variables names in the model and corresponding quantities.

Variable	Associated protein
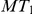	Membrane Type I Matrix MetalloProteinase (MT1-MMP)
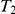	Type II Tissue Inhibitor of MetalloProteinases (TIMP2)
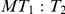	The MT1-MMP/TIMP2 complex
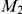	Type II Matrix MetalloProteinase (MMP2)
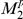	The proenzyme of MMP2 (pro-MMP2)
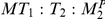	The MT1-MMP/T2/M2P complex
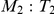	The MMP2/TIMP2 complex
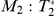	A stable isoform of the MMP2/TIMP2 complex
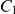	Type I collagen
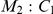	The MMP2/Collagen I complex
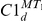	Collagen I degraded by MT1-MMP
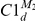	Collagen I degraded by MMP2

**Table 2:** The equations for the system.

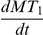	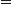	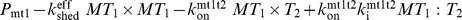
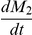	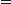	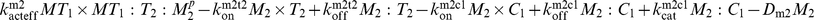
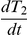	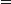	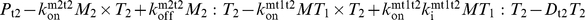
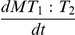	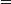	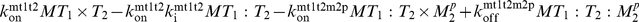
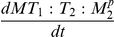	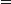	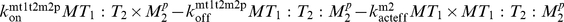
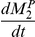	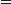	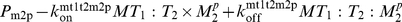
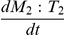	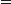	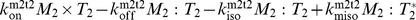
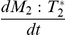	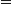	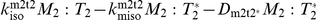
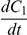	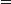	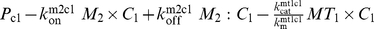
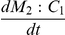	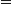	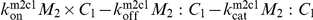
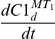	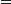	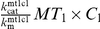
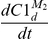	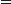	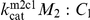

**Table 3:** Nominal values for the parameters.

Kinetic constants				Production and degradation terms	
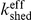	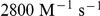	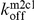	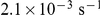	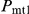	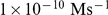
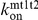	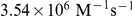	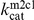	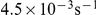	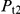	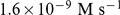
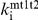	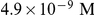	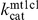	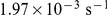	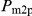	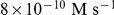
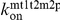	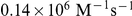	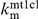	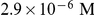	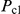	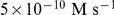
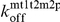	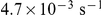	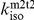	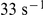	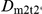	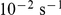
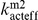	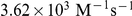	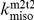	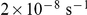	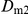	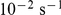
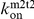	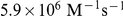	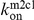	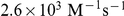	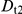	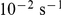
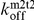	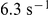				

Setting production and degradation terms to 0 yields the (closed) system described in [23].

### Quantitative insights on transient behaviors based on a simple predicate

**Figure 2:**
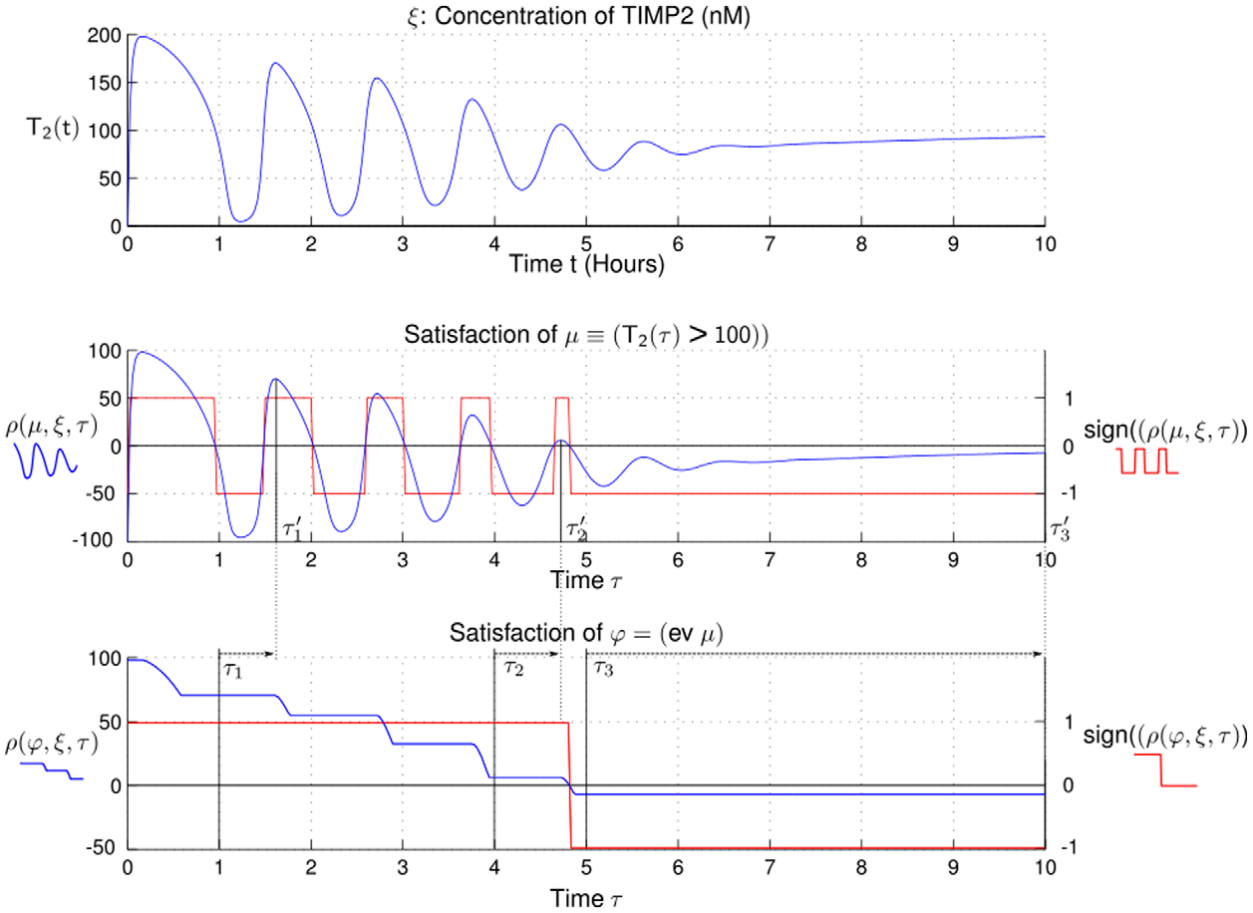
Illustration of a simple formula involving the *eventually* operator. From the behavior on the upper panel we evaluate the quantitative (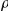) and boolean (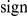) satisfaction of the predicate 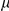 and the formula 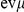. Each plateau corresponds to the amplitude of the highest peak in the future. The value of 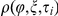 for 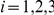 is given by 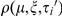 which is equal to 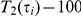. The formula is satisfied at times 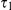 and 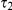 but the satisfaction is more robust for 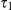 while it is weakly falsified at time 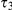.

As a first illustration of our approach, we provide a quantitative analysis of a transient and damped oscillatory behavior using a simple temporal formula involving the “eventually” operator introduced in the Methods Section (see Figure 2). We simulated the evolution of the TIMP2 concentration, noted 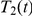, for given parameter values of the system that gives rise to damped oscillations around a 100 nM concentration value. We define a predicate 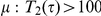 and the formula 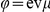, then observing their qualitative and quantitative satisfaction for different values of 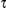. Because of the oscillations, 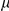 alternates between satisfaction and non satisfaction of the predicate until 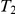 stabilizes below 100 nM slightly before 5 hours. In other words, 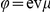 remains true before 5 hours and false afterwards. Because of the damped oscillations however, the quantitative satisfaction of 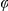, i.e., the function 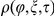, is decreasing with a staircase shape, each step corresponding to the value of the peak (maximum) ahead of 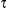.

### Discriminant analysis of the TIMP2 activation/inhibition switch

As outlined in the introduction, one key feature of the regulatory properties exhibited by the MT1-MMP, TIMP2, MMP2 biochemical network is the switching capabilities of TIMP2 on the pro-MMP2 activation. This switch between activation and inhibition of MMP2 production depends on the concentrations of MT1-MMP and of the intermediate trimer MT1-MMP/TIMP2/pro-MMP2. Thus, this switching mechanism can be quantitatively analyzed from numerical integration of the differential system of Table 2 when considering as initial conditions increasing concentrations of TIMP2 for a given pair of MT1-MMP and pro-MMP2 initial values.

#### Associated predicates and formulas

In our framework, such analysis can be conducted by defining a simple predicate that defines the percentage of activated MMP2 at time 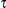 (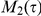) with regard to the initial pro-MMP2 concentration (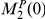):
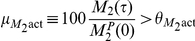
We defined an uncertain parameter set 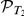 for which only 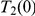 varies in a given range and considered the quantitative satisfaction of 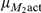: 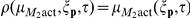 for 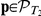 for different times 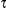.

**Figure 3:**
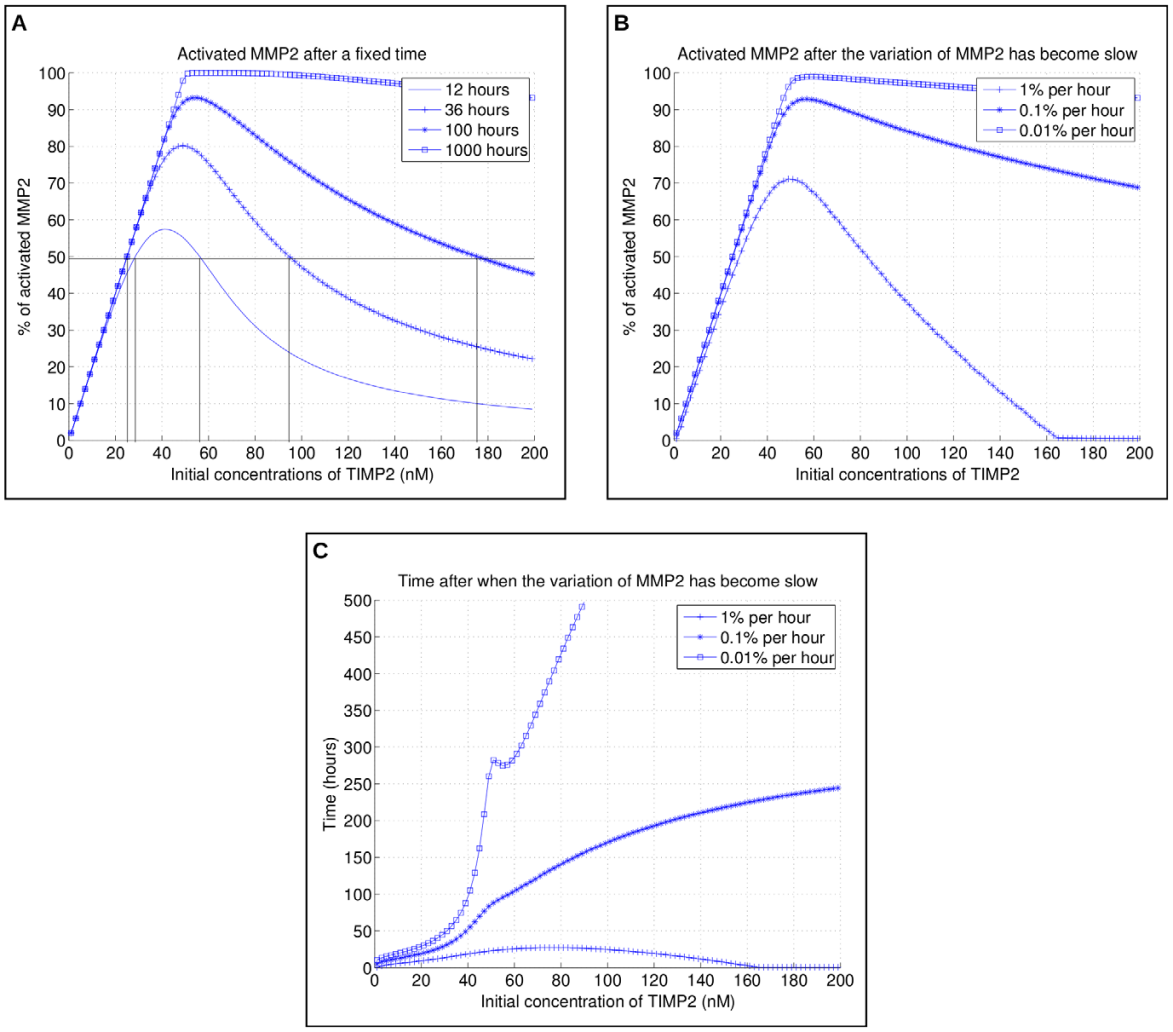
Profile of second enzyme (MMP2) production. We focus here on the activation cascade of pro-MMP2 enzyme, i.e., we exclude shedding and collagen degradation from the model. The initial concentrations of MMP-MT1 is 60 nM and initial pro-MMP2 is 50 nM, which explains the qualitative switch in **C** for initial TIMP2 greater than 50 nm. **A** Activated pro-MMP2 in percentage of initial quantity after different fixed times - the curve corresponding to 12 hours reproduces a result from [23]. The asymptotic behavior for times going to 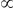 is, as suggested by the curve corresponding to 1000 hours, a linear activation from 0 to 50 nM and a plateau at 100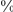 activation for initial TIMP2
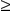
50 nM. **B** Activated MMP2 after the variation of MMP2 has reached different low rates. For initial concentration of TIMP2
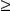
160 nM, the activation rate is never above 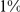, hence the percentage is 0. **C** Time after when the variation of MMP2 has reached the different low rates. The non-monotonic behavior for 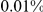 per hour above 50 nM suggests there is an optimum value for which activation is eventually (asymptotically) complete while the speed of the process at its beginning is maximized.

Using 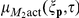 with 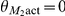, the result can be presented as a plot with initial TIMP2 concentrations along the 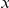-axis and the percentage 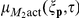 of active MMP2 formed at time 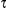 on the 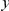-axis. Assuming that a nearly steady-state was reached after 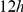, such plot was obtained by Karagiannis and Popel (2004) for TIMP2 concentrations in the range [0–200 nM]. A typical bell-shaped curve provided in [23] is presented in Figure 3 for simulations conducted with initial concentrations of 60 nM and 50 nM of MT1-MMP and pro-MMP2, respectively. A maximum value 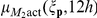 of almost 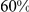 was obtained for initial TIMP2 concentration of roughly 45 nM. For larger initial TIMP2 concentrations, a marked decrease of MMP2 production takes place, down to a basal value 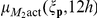 of around 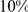 for initial TIMP2 concentration of 200 nM. This bell-shaped curve evidences the activation/inhibition switch controlled by TIMP2 initial concentrations. However, such appreciation of the inhibitory effect appears far less strong if the 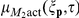 value is computed for larger time scale. We plotted on Figure 3-**A** the curves computed for 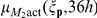, 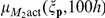 and 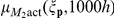, i.e., for transient regimes that are considered to be longer than the original 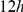 boundary. The activation range, i.e. the ascending parts of the curves, reveals only a slight shift of the TIMP2 concentrations corresponding to increasing maximal production of MMP2. On the contrary, the curves exhibit rather different profiles in the inhibition range, with the inhibitory effect linked to increasing TIMP2 concentrations being slowed down. Thus, a biased estimation of the duration of the transient mode may lead to very imprecise estimation of the biochemical network capabilities in terms of MMP2 enzyme production. For example, verifying the predicate 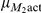 with 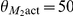 to estimate the range of TIMP2 initial concentrations for which expected percentage of active MMP2 is larger than 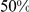 will give around [28 nM–56 nM] for 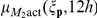, but around [25 nM–175 nM] according to 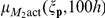 (see Figure 3 **B**). For biological processes, like angiogenesis, that strongly depend on MMPs activity on time scale corresponding to several days [37], [38], such differences are quite meaningful.

#### Evaluating uncertainty linked to transient dynamics

The above results indicate that the MMPs enzymatic system is still in transient mode after 12 hours. Unfortunately, there is no straightforward method to predict the duration of the transient modes. As briefly introduced above on a simple example, our approach provides a way to precisely quantify the network transient dynamics by monitoring the convergence rate using an appropriate predicate. This later specifies that a steady-state is almost asymptotically reached if the variation rate of the considered concentration falls below some small imposed value. More precisely, we define
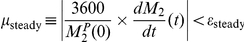
If 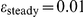 and 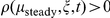, the rate of activation of 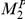 is less than 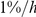 of the initial quantity of 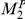. Then, we define
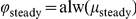
The always operator ensures that when 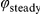 is satisfied, the rate of activation will then always remain below this threshold for larger times. We plot in Figure 3-**B** such pseudo-asymptotic steady-states for the parameter and variable values we already used in Figure 3-**A**. Both figures are very similar for small initial concentrations of TIMP2, but the determined proportion of activated MMP2 is significantly higher in Figure 3-**B** than in Figure 3-**A** in the decreasing part of the curves. For example, at 100 nM TIMP2, assuming a quasi-steady-state after 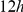 gives a computed value for MMP2 of close to 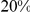, while this value doubles to almost 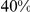 if considering that a quasi-steady-state is reached for variation rate lower than 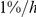 (Figure 3 **B**). Accordingly, the earliest time for which the system has reached a steady state is given by:
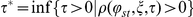
and by construction, the quantity 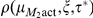 is the ratio of activated MMP2 obtained at this moment. We plot on Figure 3 **C** 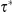 and 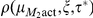 for different values of 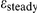.

#### Analysis of the entire enzymatic network

**Figure 4:**
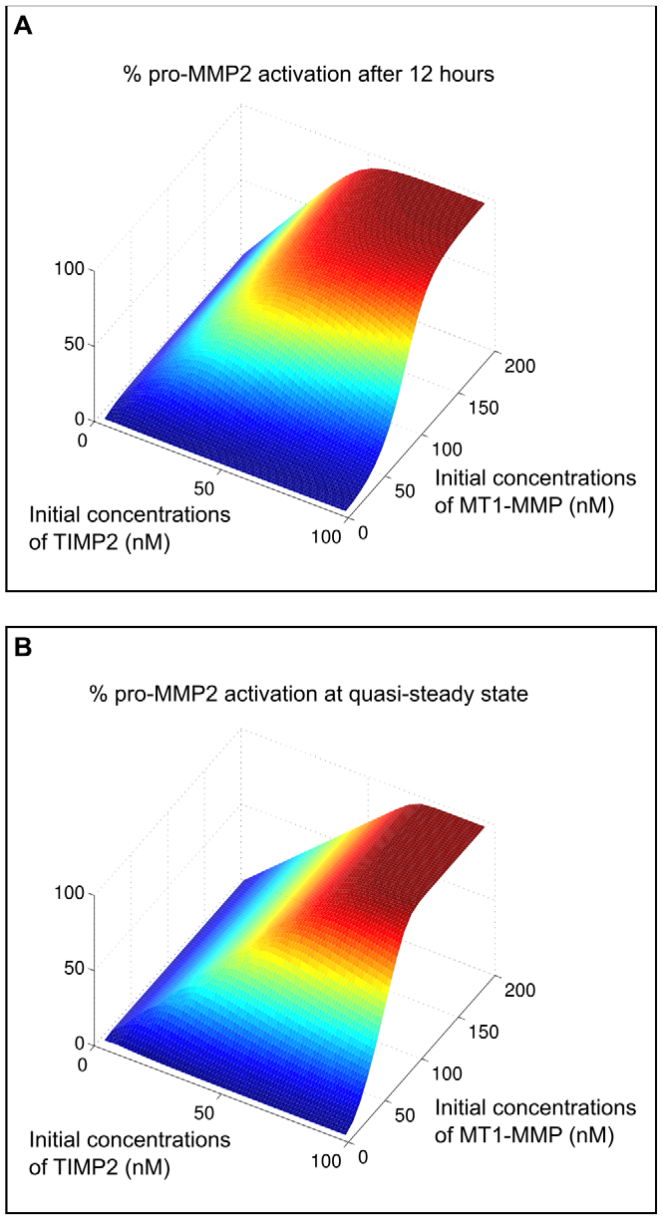
Activation/inhibition switch of MMP2 production. Here, we consider the full model of [23] including collagen degradation and ectodomain shedding of MMP-MT1. We computed the percentage of activated MMP2 depending on TIMP2 and MMP-MT1 after 12 hours **A** and after the system has reached a quasi-steady state **B**.

The above simulations were obtained by focusing on a subsystem of the model that only considers the activation of MMP2. Considering now ectodomain shedding of MMP-MT1 (the reaction by which the membrane metalloproteinases MT1-MMP proteolytically cleave themselves, secreting their catalytic domain, 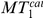) and actual collagen degradation (i.e. 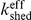 and 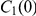 non zero, see [23]) together with varying the initial conditions of the proenzyme pro-MMP2 and by varying MT1-MMP with TIMP2, we can perform numerical simulations that express quantitatively as a 3D diagram the amount of initial pro-MMP2 that is transformed into the active MMP2 enzyme. We performed experiments for fixed-time (12 hours) and until a quasi-steady state is reached, as monitored by a variation rate of less than 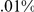 (see Figure 4). We observe that for higher initial concentrations of MMP-MT1, the activation is monotonic with the initial concentration of TIMP2 whereas for a range of lower initial quantities of MMP-MT1, TIMP2 become inhibitor at higher doses. The optimal relation for activation efficiency between initial MMP-MT1 and TIMP2 can be obtained by following the ridge on the computed surfaces. As expected, we obtain different results depending on whether the system is still in a transient regime or not (Figure 4 **A** and **B**, respectively).

#### Assessing the predictive power of the model

In [39], processing of pro-MMP2 was observed by gelatin zymography for increasing concentrations of TIMP2. In this experiment, a reduced amount of MT1-MMP was used down to a level for which pro-MMP2 processing occurs only weakly, leading to an intermediate, i.e. not fully activated, MMP2 form. The corresponding gelatinolytic band of this intermediate form was used as a reference densitometry measurement 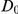. The densitometry analysis then showed an increase of the intermediate form density 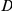 with the addition of TIMP2, indicating an enhancement of the processing of pro-MMP2 by TIMP2 up to a threshold concentration of TIMP2 above which TIMP2 action becomes inhibitory. This inhibitory switch has been clearly evidenced experimentally by plotting the ratio 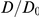 against the amount of TIMP2 in a range 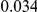 ng–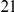 ng (Figure 3 **B** in [39]). In order to evaluate qualitatively and quantitatively the predictive power of the model, we performed a similar but virtual “experiment” by simulating pro-MMP2 processing for increasing concentration of TIMP2.

As an indicator of TIMP2 activity, we computed the ratio of the concentration of activated MMP2 after 1 hour over the concentration obtained when TIMP2 is absent for small amount of MT1-MMP of 41.7 nM processing 1.39 nM of pro-MMP2, these values corresponding to the 20 ng of MT1-MMP and 1 ng of pro-MMP2 used in Kinoshita et al experiments. A difference with the experimental setting in [39] is that our model does not include MMP2 activation by MT1-MMP alone. To account for this difference, we performed the simulations with a small initial quantity of the complex MT1-MMP/TIMP2 of 7 nM which ensures that the reference activation of pro-MMP2 in the absence of TIMP2 is non null.

**Figure 5:**
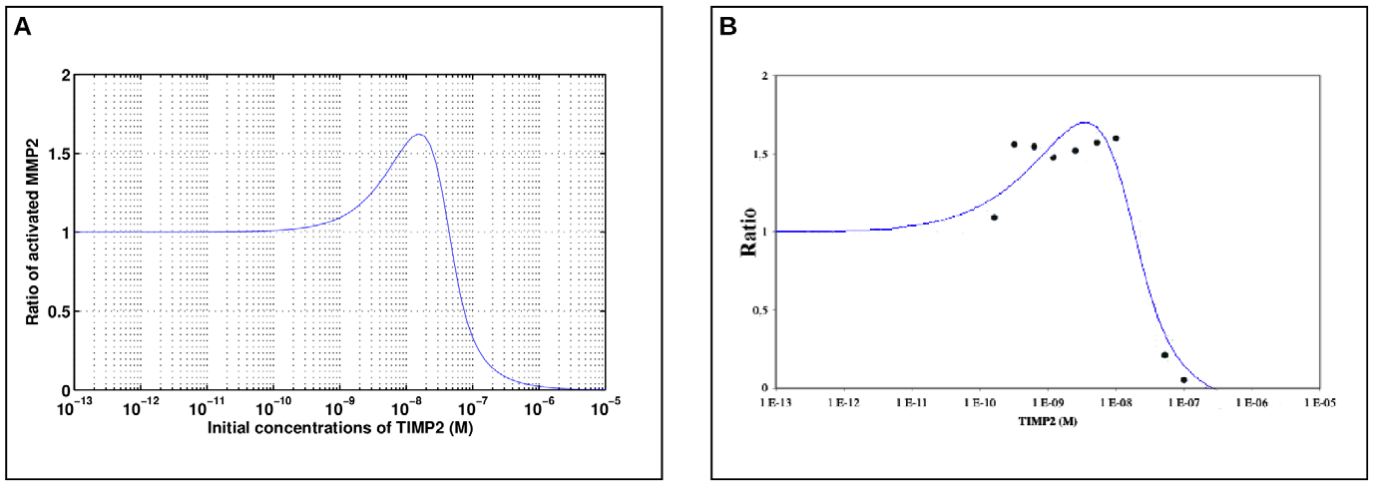
Asserting the predictive power of the model. **A**: ratio of the concentration of activated MMP2 after 1 hour over the concentration obtained when TIMP2 is absent as simulated by our model. Initial concentration of MT1-MMP proMMP2 was 41.7 nM and 1.39 nM respectively , these values corresponding to the 20 ng of MT1-MMP and 1 ng of proMMP2 used in Kinoshita et al experiments [39]. The initial quantity of the complex MT1-MMP/TIMP2 was set to 7 nM to ensure that the reference activation of proMMP2 in the absence of TIMP2 is non null. **B**: experimental measurements of the activation replotted from [39].

The so-simulated TIMP2 switch effect has been plotted in Figure 5 **A**. We started from very low values of TIMP2 concentrations, from which the ratio value remains close to one in a large range of TIMP2 concentrations, as in [39]. Then the ratio increases, with an overall simulated plot that compares very satisfactorily, both qualitatively and quantitatively, with the experimental data reported in Figure 3 **A** of [39]. For the sake of comparison, these data have been re-plotted here in Figure 5 **B**, using as 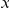-axis the corresponding molarity range of TIMP2.

### Discriminant analysis of the relative contribution of MT1-MMP and MMP2 to collagen degradation

As stated in the introduction, both MT1-MMP and MMP2 participate to collagen proteolysis. Thus, our quantitative analysis of the enzymatic network can be proved useful in gaining insight into the *respective contribution* of MMP2 and MT1-MMP on the proteolysis of collagen in the presence of TIMP2. Studying such enzymatic synergism should provide precise boundaries on the initial values and concentration ratios of pro-MMP2, MT1-MMP and TIMP2 under which the MMP2-dependent proteolysis is larger (or lower) than the proteolysis controlled by MT1-MMP.

To answer these questions, Karagiannis and Popel [23] compute a proteolysis diagram that gives the initial TIMP2/pro-MMP2 and MT1-MMP/TIMP2 ratios under which the proteolysis induced by MMP2 is maximal at different times (Figure 7-b of [23]). After 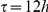, they found that the collagen proteolysis is mostly carried out by MT1-MM for low pro-MMP2 concentrations, while a proteolytic balance or enzymatic synergism between MT1-MMP and MMP2 comes into play at higher pro-MMP2 concentration levels.

**Figure 6:**
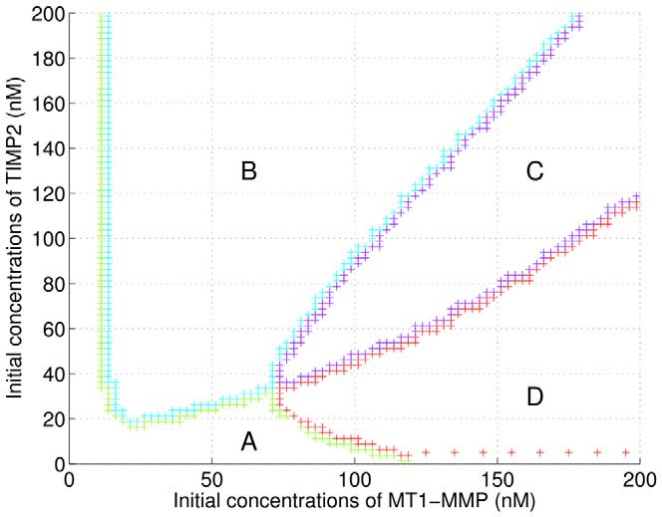
Values of initial pro-MMP2 and TIMP2 exhibiting synergism. In region C, we have 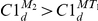 and 90 percent of the collagen is degraded after 12 hours (i.e. 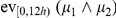). In D, we have 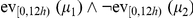, i.e., most of the collagen is degraded but mainly by the action of MMP-MT1. In B, we have 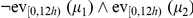 and in A 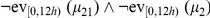. In both cases A and B, the system does not manage to degrade 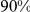 of the collagen before 12 hours.

In this study, we take benefit of the convenient predicate-based framework we developed to get quantitative insights into the relative contribution of MT1-MMP and MMP2 on collagen proteolysis on a more general and more accurate basis. Indeed, our approach allows to formulate a combination of constraints imposed on -(i) the amount of collagen that has been degraded after a given time and -(ii) the respective contribution of any of the two enzymes onto collagen degradation. As a practical illustration, we define two predicates:

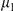
-the proportion of collagen degraded by both proteases at a given time is greater than 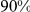: 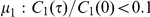

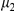
-the amount of collagen degraded by MMP2 is at least equal to the amount of collagen degraded by MT1-MMP : 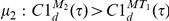

The results we obtained are summarized on Figure 6 for varying initial concentrations of TIMP2 and MT1-MMP in the range [0 200 nM], with the percentage of degraded collagen being evaluated after 12 h. The different regions of the diagram correspond to initial values of TIMP2 and MT1-MMP such that: 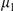 only is satisfied (region D), 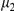 only is satisfied (region B), 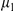 and 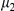 are both satisfied (region C), 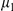 and 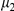 are not satisfied (region A).

### Global robustness analysis

In the previous sections, we analyzed the enzymatic network dynamics by varying a small number of parameters, keeping a fixed value for the others. In this section, we perform a more global analysis of the system by considering a parameter set 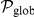 of varying initial concentrations of the four variables 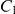 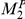, 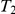 and 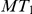 between 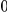 and 200 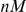. We also varied the fifteen kinetic constants within 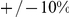 of their nominal values. The high-dimensionality of the resulting search space (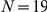) prevents the construction of precise boundaries as performed in the previous section so we used the quasi Monte Carlo approach.

**Figure 7:**
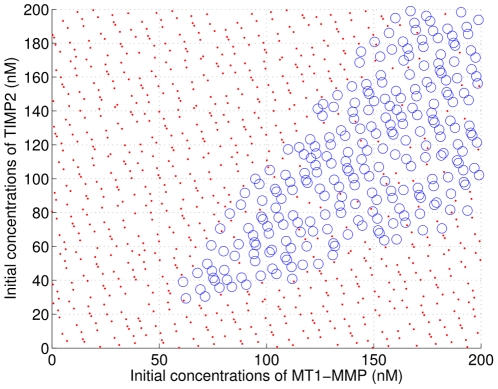
Global robustness analysis of synergism. Each red point corresponds to a trajectory for which 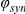 is not satisfied and each green circle corresponds to a trajectory satisfying 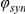. Note that the 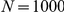 samples parameters represented on this figure are also uniformly distributed in the other dimensions of the parameter space corresponding to varying initial concentrations of collagen 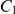 and varying values of the kinetic chemical constants with 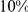 of their nominal values.

We sampled the set of possible values of initial conditions and parameters with 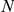 vectors evenly distributed in the parameter space. For each of these vectors, we generated a trajectory and observed a property of interest. For instance, we considered the synergism between degradation of collagen by MMP-MT1 and MMP2 as being characterized by the predicate:
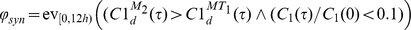
Among the 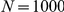 sampled trajectories, we found that 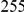 satisfy 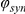. On Figure 7 the point associated with each simulated trajectories has been marked by an open circle if the trajectory satisfies 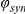 and a plain dot otherwise. Interestingly, the shape of the region in which the property 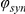 is very similar to the shape of region C in Figure 6. Thus our approach provides a strong support for assessing the robustness of the enzymatic network with respect to the 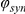 property since -(i) the volume 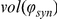 where 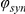 is true is significant and -(ii) its shape is preserved even when varying as many as 19 parameters and initial conditions.

**Figure 8:**
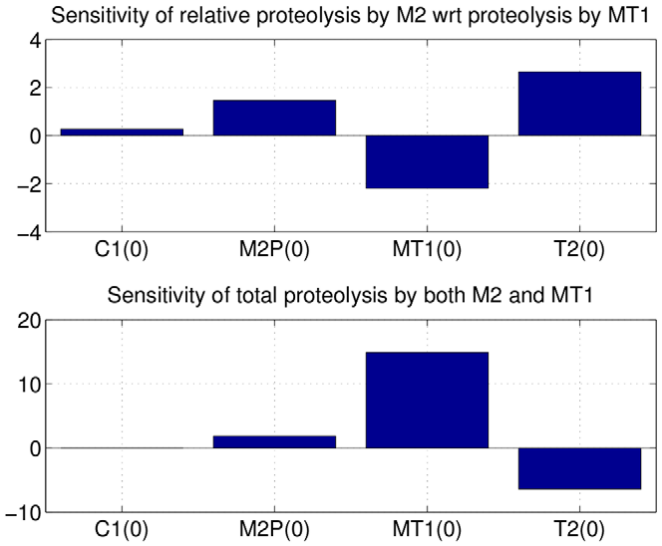
Global sensitivity analysis of proteolysis with respect to initial concentrations after 12 h. These histograms were obtained by applying the global sensitivity formula (8) for N = 1000 samples in the parameter set 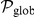 where 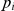 is replaced by the initial concentrations 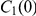, 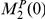, 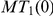 and 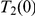. The top histogram indicate that TIMP2 has globally an activation role for proteolysis controlled by M2-MMP, but a globally negative influence on the total degradation of collagen (bottom histograms), while 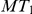 has a globally positive influence on it.

Finally, we performed a global sensitivity analysis with respect to the proportion of collagen degraded after 12 hours (reusing predicate 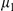) and with explicit consideration of the respective contributions of MT1-MMP and MMP2 in the degradation process (using predicate 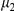) by averaging local sensitivity analysis on the same sample of trajectories as above. We measured the relative variations of 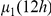 and 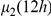 with respect to variations in 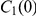, 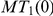, 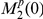 and 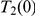 (using formula (8)). The results are represented on Figure 8. They are consistent with the rest of the analysis: increasing the initial concentrations of MT1-MMP enhances collagen proteolysis, whereas TIMP2 has globally an inhibitory action on the degradation process. As expected, increasing TIMP2 favors the activation of pro-MMP2 and thus the proteolysis by MMP2 against that controlled by MMP-MT1.

### Discriminant analysis with regard to the existence of oscillatory proteolysis

The biochemical system we analyzed in the previous sections is a closed system that admits only trivial asymptotic dynamics: depending on the initial conditions, the concentrations of the species eventually converge toward different *stable steady-states*. Therefore, we extended our analysis by considering the dynamics of MMP2 and MT1-MMP in the presence of TIMP2 in conditions closer to *in vivo* situations, where secretion/degradation processes come into play. Thus, this section deals with the analysis of an open enzymatic system in which secretion of MT1-MMP, pro-MMP2 and TIMP2 have been introduced, together with collagen bio-synthesis. In addition to proteolysis, degradation of MMP2, TIMP2 and of the binary complex MMP2:TIMP2* have been considered. The non-linearity of the associated differential system may generate oscillations of the model variables if the open system steady-state becomes unstable due to coupling of MT1-MMP, MMP2 and TIMP2 pathways. This corresponds to a bifurcation, i.e. to a qualitative change in the system dynamics. The different regions of the parameter space where different types of oscillatory behaviors would exist have to be bounded. Additionally, the sensitivity of such oscillatory behaviors to changes in parameter values has to be assessed in order to gain significant and quantitative information on the range of parameter values producing such oscillations as well as on the variation of oscillations shape (amplitude, period, etc) over this range. Let us recall that exact detection of periodic trajectories generated by deterministic nonlinear systems is not a trivial problem. Classical techniques include Poincaré's first return maps, bifurcation analysis using continuation techniques, etc. Most of these approaches are based on the study of the asymptotic behavior and aim at proving the existence of a (stable) limit cycle beyond some threshold of a considered bifurcation parameter. A simple example of bifurcation in which oscillations arise is the so-called Hopf bifurcation. Performing a standard bifurcation analysis of the biochemical model based on system linearization in a neighborhood of steady-states would be straightforward in principle but would involve rather lengthy algebraic efforts. Following the approach we propose here, the oscillatory behavior of the enzymatic network was explored by defining temporal constraints that are specified by the characteristics (amplitude, period, etc) of the oscillatory behaviors we are looking for.

#### Approximate oscillations detection

Here, we keep the formal framework of the preceding section and make use of temporal constraints on the transient behavior that must be satisfied in order to explore the model capability to exhibit sustained oscillations. For instance, looking for oscillatory behaviors in the concentration of TIMP2, we required first that it remains under a certain threshold, i.e., that the formula 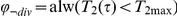 holds. Second, we specify that its value eventually always alternates between periods when the concentration increases at a rate above some strictly positive value 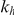 and periods when the concentration decreases at a rate below some strictly negative value 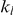:
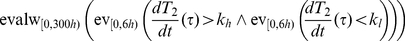
(25)


Using this formula, as well as the one obtained by substituting 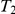 for 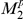, we performed a systematic analysis to characterize the domain of existence of the oscillatory regime for TIMP2 and pro-MMP2 for different values of production fluxes. Starting from a given set of initial conditions, four different types of dynamical behaviors were characterized according to the chosen model parameters:

1. A monotonic increase of TIMP2 and pro-MMP2;
2. An asymptotic convergence toward a stable steady-state;
3. An asymptotic convergence toward a stable limit cycle for all of the model variables, except pro-MMP2 that increases;
4. An asymptotic convergence toward a stable limit cycle, with self-sustained oscillations of all the model variables.

**Figure 9:**
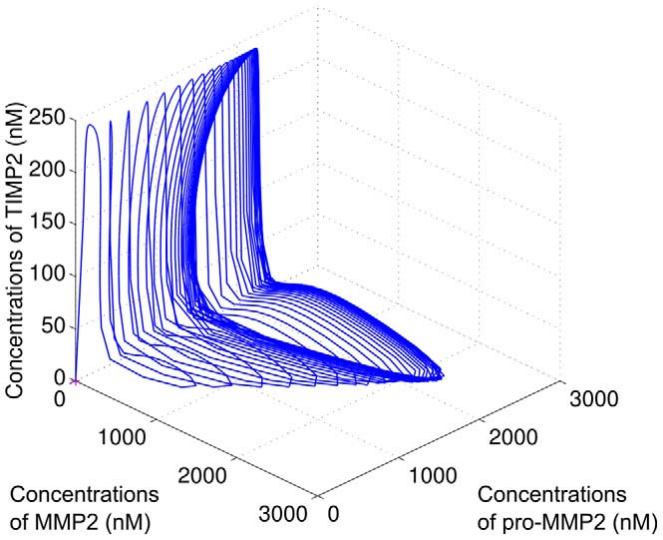
Limit cycle in the space 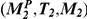. One can observe that the phases of 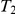 and 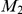 are in opposition: 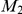 is low when 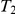 is high and vice-versa.

**Figure 10:**
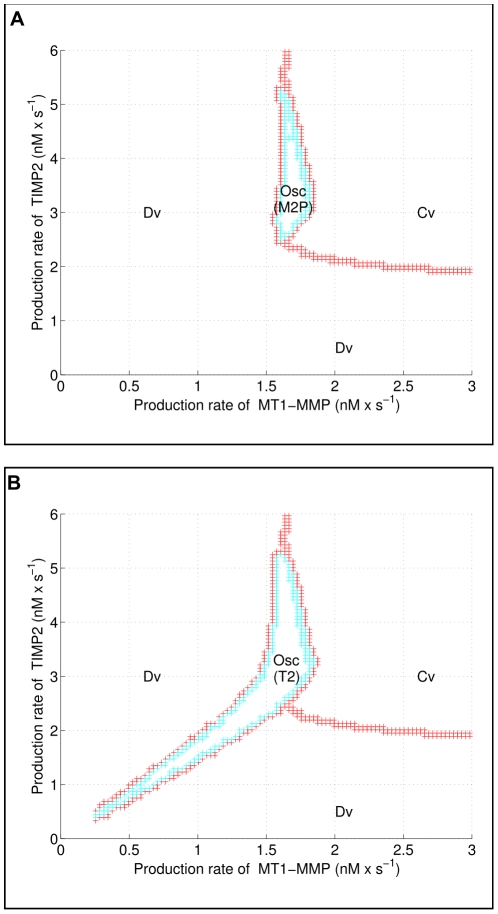
Two maps of different regimes. Region Cv: convergence toward a steady state, Region Osc M2P: 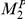 and all other variables oscillate, Region Osc (T2): oscillations of 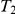 and other variables but increase of 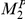 for the tail-like part of the region, Region Osc (T2)), Region Dv: increase of 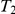 and 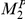.

A trajectory converging toward a limit cycle is plotted in Figure 9. Figure 10 represents the 2D-bifurcation diagram that has been constructed by taking as bifurcation parameters the production rates of MT1-MMP and TIMP2, i.e., 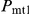 and 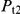, respectively (see Table 3). Our analysis distinguishes four different regions, corresponding to the cases defined above.

**Figure 11:**
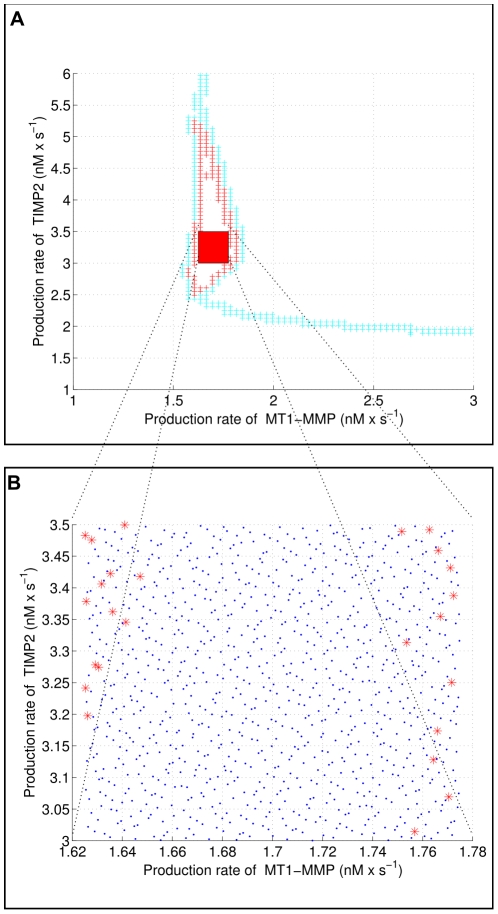
Assessing the robustness of oscillations. On **A**, we extracted a subset of the region where the oscillatory regime is observed and augmented it with uncertain parameters. **B** We sampled the resulting parameter space with 1000 quasi-random samples. The blue dots and the red stars correspond to parameter values generating oscillatory and non oscillatory trajectories, respectively. Only 25 out of the 1000 sample trajectories do not exhibit oscillations.

To assess the robustness of the oscillatory regime inside the region found in the 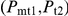 parameter plane in the previous analysis, we defined a subset of this region enlarged by adding a 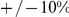 variation on the other parameters, then performing a quasi-random sampling with 1000 trajectories in the resulting parameter set. The results are given in Figure 11. Overall, the oscillatory regime is preserved despite the variations in the parameters, except for 25 values distributed on the border of the region projected in the 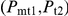 plane, which means that the oscillatory regime is globally robust for these ranges of production fluxes 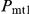 and 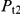.

## Discussion

In this paper, we proposed a temporal-logic based methodology for the robustness analysis and behavior discrimination of enzymatic reaction networks. Beginning from early 2000s, different approaches aiming at bringing formal methods and reasoning from pure computer science and model-based design of engineered systems to biology have been proposed. The usual idea is to leverage advanced abstraction techniques and tools that have been developed in the original context of formal methods and apply them to biological systems. To do this, the most important step is to get an input model which is compatible with the methodology to be used. Thus a strong emphasis is put either on the development of *modelling languages* which are both adapted to the description of biological processes and amenable to a formal analysis with existing techniques (see, e.g., [40], [41]), or on the approximation of existing models into such adapted formalism (e.g. in [12] and other works by these authors, genetic and metabolic networks are approximated with piecewise affine systems and Boolean networks). Our work takes a different approach which is both simpler and more ambitious. It is simpler in that we do not question the description language used for the system and neither propose a new one. Instead, we consider the modelling formalism of differential equations, which has known limitations but is already familiar to biologists and to a certain extent commonly used by them, and focus on the *property language*, which is adapted to trajectories simulated from an ODE model. It is more ambitious in that applying formal methods such as model-checking to high-dimensional non-linear differential equations is notoriously difficult, and existing methods targets mostly Boolean or discrete models (a recent illustration is [42]). This is why we restrict ourselves to a simulation-based approach which do not always provide formal guarantees because it is subject to numerical errors, but will provide answers as soon as a simulation can be performed. This is also why quantifying robustness is an important part of our approach. Closer to our work, [33] define a validation degree for a trajectory and a formula which is similar to our robust satisfaction function. However, these authors make use of a discrete-time logic that cannot deal with continuous time intervals. Moreover, the computational cost of their validation degree, in the worst case, scales exponentially with the length of the trajectory and the formula, while it remains polynomial in our approach [18]. To the best of our knowledge, our approach is the only one which is quantitative and based on signal temporal logics, i.e. adapted to continuous time-varying trajectories.

We used our methodology to analyze the dynamics of an enzymatic network describing the activity and regulation of the metalloproteinases MMP2 and MT1-MMP by a common inhibitor, TIMP2.

Our analysis provides a detailed and quantitative analysis of the switch-like effect of TIMP2 concentration on the dynamics of MMP2 production from the latent pro-MMP2 form of the enzyme. With respect to the earlier analysis of [23], we showed that an underestimation of the transient period may lead to inaccurate interpretation of the TIMP2 switching activity. Furthermore, we showed that our framework can be used to detect automatically the approach of steady states, thus allowing a more complete systematic analysis of proteolytic and remodelling processes taking place over several days, as observed for example during *in vitro* angiogenesis. Interestingly, we showed that our framework is flexible enough to formalize experimental protocols and associated results not considered during the phase of model construction, thus allowing to analyze model predictions. This point has been illustrated by simulating very satisfactorily the experimental work of Kinoshita et al. [39] on TIMP2 activity, which has not been considered in the model development conducted in Karagiannis and Popel's paper [23]. Additionally, our approach provides a clear and systematic analysis of the synergistic action of both MMP2 and MT1-MMP onto extracellular collagen matrix proteolysis. This synergy is indeed a key regulatory process that enhances the proteolytic activity of migrating cells acting upon the extracellular environment barriers.

In a second part of this work, we extended the original model of [23] by considering an open network system, in which enzyme production by cells has been taken into account. Interestingly, we identified an oscillatory regime that highlights the multi-functional role of TIMP2. More precisely, the sequestration of TIMP2 into complexes involving both MT1-MMP for MMP2 activation and MMP2 itself for MMP2 inhibition generates the feedback loop that destabilizes the enzymatic network steady-state. Since the network can exhibit different dynamical regimes depending on the values of the model parameters, we took benefit of our temporal logic approach to characterize the subdomains of the parameter space that are associated to each regime. By formalizing the characteristic properties of a given regime as temporal logic constraints, we were able to compute accurately the boundaries separating regions corresponding to different dynamical regimes in a two dimensional plane formed by the production fluxes of TIMP2 and MT1-MMP. By sampling the parameter space with a Quasi Monte Carlo algorithm, we showed that these boundaries remain valid even when considering uncertainty on all other network parameters, and that the oscillatory domain is rather large within the parameter space. This suggests that oscillatory dynamics in the MMP2 activation cascade might be encountered *in vivo*, with the MT1-MMP/MMP2/TIMP2 enzymatic network possibly acting then both as a switch and as an oscillator under different environmental conditions. However, such predictions have some limitations since our analysis does not explicitly consider the confinement of active MT1-MMP at the plasmic membrane. Nevertheless, our analysis of this metalloproteinase network gives new insights regarding the parameter domains that are crucial for the proteolytic activity of these interacting enzymes, together with a quantitative characterization of the reaction network sensitivity to perturbations of reaction rates or affinity constants. The temporal logic approach developed here can clearly be applied to other biochemical networks containing regulatory structures, especially in order to identify the different dynamical regimes and to characterize the feasible regions in parameter space where the robustness of a given behavior has to be assessed.

## Supporting Information

Text S1 **Implementation: the breach toolbox.**
(TXT)

Text S2 **Note on computing local sensitivity for satisfaction function.**
(PDF)
