# Supplementary material for: Robustness Analysis and Behavior Discrimination in Enzymatic Reaction Networks
Source: PLoS One. 2011 Sep 27;6(9):e24246. doi: 10.1371/journal.pone.0024246 (PMC3181259; doi:10.1371/journal.pone.0024246)
Supplement: Text S2 — Note on computing local sensitivity for satisfaction function. (PDF) [file pone.0024246.s002.pdf]

Note on Computing Local Sensitivity for Satisfaction Function

This technical appendix describes briefly how to compute the local sensitivity of the robust satisfaction function for a formula φ. If φ is a simple predicate μ, this is achieved by applying the chain rule to get the derivative of the function $\mu(\xi, \tau)$, as follows. To simplify, let us assume that ξ depends only on a parameter p. We have

$$\frac{d\rho(\mu, \xi_p, \tau)}{dp} = \frac{d\mu(\xi_p, \tau)}{dp} = \frac{\partial \mu}{\partial \xi_p}(\xi_p, \tau)\ \frac{d\xi_p}{dp} \qquad (1)$$

where the second term in the multiplication is provided by the sensitivity function. For formulas φ involving Boolean and temporal operators, the function $\rho(\varphi, \xi_p, \tau)$ is obtained as a result of maximum and minimum operations on the predicates appearing in φ so that at the end there is at least one pair of (predicate, time) $(\mu_i, \tau_i^* \geq \tau)$, such that $\rho(\varphi, \xi_p, \tau) = \mu_i(\xi_p, \tau_i^*)$. If this pair is unique, then the derivative of ρ at τ is simply the same as the derivative of μ_i at τ_i^*, i.e., $\frac{d\rho}{dp_i}(\varphi, \xi_p, \tau) = \frac{d\rho}{dp_i}(\mu_i, \xi_p, \tau_i^*)$. Otherwise, if there is another pair (μ_j, τ_j^*) such that $\rho(\varphi, \xi_p, \tau) = \mu_i(\xi_p, \tau_i^*) = \mu_j(\xi_p, \tau_j^*)$ then ρ may not have a derivative in (p, τ). Indeed, we know that the derivative with respect to x of $\max(f(x), g(x))$ (or $\min(f(x), g(x))$ may not be defined when $f(x_0) = g(x_0)$, even if both have a derivative in x_0. In this case, though, ρ has a left-derivative and a right-derivative which can be computed, as discussed in [1].

References

1. Donzé A, Maler O (2010) Robust satisfaction of temporal logic over real-valued signals. In: Chatterjee K, Henzinger TA, editors, FORMATS. Springer, volume 6246 of *Lecture Notes in Computer Science*, pp. 92-106.
